# Impact of oral precancerous lesions on oral cancer development in patients with oral lichen planus: a retrospective cohort study of 318 oral lichen planus patients

**DOI:** 10.3389/froh.2025.1560600

**Published:** 2025-03-06

**Authors:** Yu-Cheng Chu, Pei-Yu Lin, Wan-Ting Huang, Hsun-Yu Huang, Chien-Chin Chen

**Affiliations:** ^1^ Department of Medical Education, Kaohsiung Veterans General Hospital, Kaohsiung, Taiwan; ^2^ Department of Stomatology, Ditmanson Medical Foundation Chia-Yi Christian Hospital, Chiayi, Taiwan; ^3^ Clinical Medicine Research Center, Ditmanson Medical Foundation Chia-Yi Christian Hospital, Chiayi, Taiwan; ^4^ Department of Pathology, Ditmanson Medical Foundation Chia-Yi Christian Hospital, Chiayi, Taiwan; ^5^ Department of Cosmetic Science, Chia Nan University of Pharmacy and Science, Tainan, Taiwan; ^6^ Doctoral Program in Translational Medicine, National Chung Hsing University, Taichung, Taiwan; ^7^ Department of Biotechnology and Bioindustry Sciences, National Cheng Kung University, Tainan, Taiwan

**Keywords:** chronic periodontitis, lichen planus, oral cancer, oral lichen planus, oral precancerous lesion, risk factor

## Abstract

**Background:**

Oral lichen planus (OLP) has been implicated as a potential risk factor for oral cancer. This study aimed to investigate the long-term determinants of oral cancer development following a diagnosis of OLP.

**Materials and methods:**

A retrospective cohort study of 318 patients with histopathologically confirmed OLP was conducted at a tertiary medical center from 1995 to 2018. Patients were categorized into two groups based on the presence or absence of oral precancerous lesions (OPLs). Baseline characteristics, underlying medical conditions, and lifestyle factors were compared between groups. The hazard ratio (HR) and the 10-year cumulative risk for oral cancer development were estimated using logistic regression and Kaplan–Meier analysis, respectively.

**Results:**

In our cohort, a total of 33 patients (10.38%) were diagnosed with oral cancer. After adjusting for confounders, OPLs (HR, 2.98), age over 50 years (HR, 4.36), chronic kidney disease (HR, 4.46), and alcohol consumption (HR, 4.23) emerged as independent risk factors for oral cancer development in patients with OLP (*P* < 0.05).

**Conclusion:**

The current study indicates that the presence of OPLs, including histologically confirmed verrucous hyperplasia and varying degrees of oral dysplasia, is associated with an increased risk of oral cancer development in patients with OLP. Further research is needed to elucidate the underlying molecular mechanisms linking OLP, OPL, and oral cancer occurrence.

## Graphical Abstract

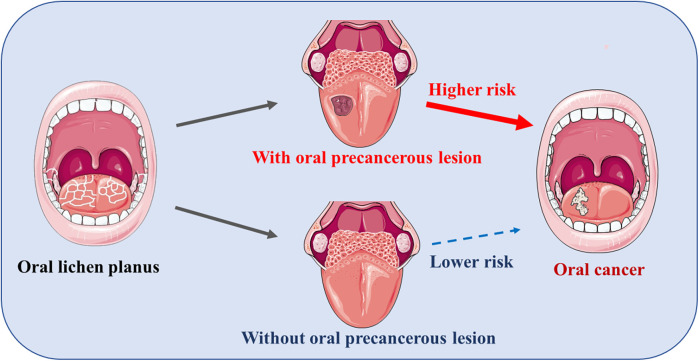

## Introduction

Oral lichen planus (OLP) is a chronic inflammatory disease characterized by T-cell-mediated immune dysfunction targeting oral epithelial cells. It affects approximately 1.27% of the global population and predominantly manifests in women aged 40–60 years (1, 2). In Taiwan, the women-to-men occurrence ratio can be as high as 4:1 or 5:1 (3). OLP is classified into three primary subtypes: hyperkeratotic (which includes reticulate, papular, and plaque/verrucous forms), erosive (which includes erosive erythematous and erosive atrophic forms), and bullous OLP (4). OLP commonly affects the lips, buccal mucosa, dorsum of the tongue, and gingiva (5). This leads to white stripes or mesh-like plaques on the oral mucosa; sometimes, oral ulcers can form. Symptoms include odynophagia, dysphagia, dysgeusia, and sensitivity to hot and spicy foods (4). If OLP symptomatic flares, first-line treatment is topical corticosteroids, which appear to be the most economical and efficacious. Topical calcineurin inhibitors are the second most cost-effective treatment. Other treatments included systemic corticosteroids, systemic retinoids, topical/systemic immunomodulators, etc. (6, 7). In addition, systematic reviews have established OLP as a potential premalignant condition of the oral cavity. Reported oral cancer incidence rates among OLP patients range from 0.44% to 2.28% (8).

A recent global report indicated approximately 370,000 new cases and 170,000 deaths attributed to lip and oral cavity cancer, with the majority of cases originating from the Asian continent (9). To prevent oral cancer, medical screening for oral precancerous lesions (OPLs) is essential. OPLs, which may progress to oral cancer, include leukoplakia (the most prevalent), erythroplakia, oral submucous fibrosis, and verrucous hyperplasia. Additionally, underlying conditions such as dyskeratosis congenita syndrome and HPV infection can increase the risk of developing oral cancer (10). Among oral cancers, oral squamous cell carcinoma (OSCC) accounts for more than 90%. Other oral cavity tumors include those of the minor salivary glands, melanomas, lymphomas, and sarcomas (11). Suspicious oral malignancies are typically diagnosed through visual inspection and a subsequent incisional biopsy (12). After diagnosis, surgical resection is the primary treatment for most oral cancers. A clear surgical margin, defined as a resection margin of at least 5 mm, is associated with the highest 5-year survival rates (13, 14). A close margin was considered 1–5 mm, while an involved margin was <1 mm and had the lowest survival rate (14). Since prevention is better than cure, researchers are committed to identifying the molecular mechanisms of OLP that lead the affected oral epithelium to malignancy.

A scoping review suggests that the carcinogenic potential of OLP may be attributed to several factors: chronic inflammation (which resembles autoimmune disease), accelerated epithelial proliferation, evasion of growth inhibitory signals, and impaired apoptosis (15). Additional key factors implicated in tumorigenesis include cigarette smoking, alcohol consumption, and hepatitis C virus infection (8). Recent studies have explored the potential of TP53 mutation screening as a biomarker for identifying OLP patients at increased risk of malignant transformation. Given the association with cellular stress, TP53 mutations may be an early indicator of neoplastic progression (16). However, large-scale, prospective multicenter studies are warranted to establish TP53 screening as a reliable prognostic tool. In addition, a recent study observed elevated CD146 protein expression in OLP, contrasting with its downregulation in OSCC. These findings suggest a potential role for CD146 in the immune response associated with OLP and the early stages of oral carcinogenesis (17). Moreover, research focusing on familial OLP, a rare condition, has revealed a heightened risk of OSCC compared to sporadic cases. Consequently, individuals with familial OLP necessitate more frequent oral cancer screening due to their increased susceptibility (18).

OLPs have been considered to be a potential precancerous lesion by the World Health Organization (WHO). Up to a third of OPLs may transform into squamous cell carcinomas (19). Our previous study also found that OLPs could be associated with higher risks of chronic periodontitis and other OPLs (including pathology-proved verrucous hyperplasia and mild, moderate, and severe oral dysplasia) (5). Particularly younger patients (<50 years) with OLP, who are supposed to be more difficult to maintain oral hygiene owing to uncomfortable oral lesions or have been exposed to inflammation for too long, have a higher incidence of periodontitis. In patients with OLP, periodontal disease, male, betel nut consumption, smoking, and candidiasis infection are all risk factors for developing OPLs (5). However, whether OLP patients with OPLs have a higher risk of developing oral cancer remains unknown. In the past 5 years in Asia, an experiment in Thailand implied OLP to be one of the most frequently observed histopathological results of clinically suspected oral cancer and OPLs (20). However, there has been no relevant research in other Asian countries, especially the Chinese population, within 5 years. Therefore, this longitudinal long-term cohort study aims to investigate further risk factors of oral cancer development of 318 OLP patients with or without OPLs in southern Taiwan.

## Materials and methods

### Patient enrollment and data collection

This retrospective cohort study was conducted in southern Taiwan, permitted by the Institutional Review Board of Ditmanson Medical Foundation Chia-Yi Christian Hospital (CYCH-IRB-2019075). A total of 318 patients with histopathologically confirmed OLP between 1995 and 2018 were included in the study. Each patient's basic characteristics, underlying diseases, and living habits were collected from their medical charts, including age, gender, OLP location, systemic disease [e.g., liver diseases, diabetes mellitus, cardiovascular disease, peptic ulcer, chronic kidney disease (CKD), hyperlipidemia, autoimmune diseases, and psychiatric diseases], alcohol drinking, betel nut chewing, cigarette smoking, periodontitis, TMJ disorder, candidiasis, skin lichen planus, and cancer.

### OLP diagnosis, oral precancerous lesion, chronic periodontitis, oral cancer diagnosis

As in our previous study, OLP inclusion criteria was a clinical and histopathologic diagnosis based on the criteria of the American Academy of Oral and Maxillofacial Pathology (AAOMP) in 2016 (21). OLP diagnoses were recorded by ICD-10 codes L43, L43.0, L43.8, and L43.9. OPLs, based on pathological diagnoses of verrucous hyperplasia and mild, moderate, and severe oral dysplasia, were adopted from patients' medical records. Periodontal disease was diagnosed using ICD-10 code K0530, with alveolar bone loss confirmed via panoramic radiographs. The initial assessment focused on interproximal tissue loss and bone loss of less than 15% on radiographs. Oral cancer was diagnosed using clinical criteria and confirmed by the oral surgeon through biopsy and histopathological examination, ensuring the accuracy and reliability of the findings.

### Statistical analysis

Mean continuous variables between groups were analyzed using the Student's *t*-test. Categorical variables between groups were analyzed using Pearson's Chi-square test and Fisher's exact test. The logistic regression model evaluated results adjusted for confounders and interactions with the hazard ratio (HR) and 95% confidence interval (CI). The 10-year cumulative oral cancer incidence rate in OLP patients was measured using the Kaplan–Meier analysis. The significant value is defined as two-tailed *P* < 0.05. All statistical analyses were conducted with SAS 9.4 for Windows (SAS Institute, Inc., Cary, NC, USA). The schematic diagram is drawn based on resources from the “https://smart.servier.com/” website.

## Results

### The differences between OLP patients with OPLs and those without OPLs

The clinical characteristics of OLP patients with and without OPLs are presented in Table 1. Initially, 318 OLP patients were acknowledged to consist of our study's criteria and divided into two groups: the OPL group (*n* = 111) and a control group (*n* = 207) without subsequent OPLs. OPLs were more prevalent in OLP patients younger than 50 years (54.95%) and male patients (85.59%). In addition, OLP patients with OPLs had higher rates of alcohol drinking, betel nut chewing, cigarette smoking, candidiasis infection, and suffering from oral cancer than those without OPLs. On the other hand, skin lichen planus rarely occurs in OLP patients with OPLs.

**Table 1:** Baseline characteristics of oral lichen planus patients with and without oral precancerous lesion.

Characteristic, *n* (%)	Total (*N* = 318)	OPL (*n* = 111)	non-OPL (*n* = 207)	*P* value*
Age (years)				
<50	131 (41.19)	61 (54.95)	70 (33.82)	<0.001**
≥50	187 (85.81)	50 (45.05)	137 (66.18)	
Location				
Buccal	235 (73.90)	87 (78.38)	148 (71.50)	0.228
Non-buccal	83 (26.10)	24 (21.62)	59 (28.50)	
Male	192 (60.38)	95 (85.59)	97 (46.86)	<0.001**
Liver diseases	60 (18.87)	20 (18.02)	40 (19.32)	0.881
Diabetes mellitus	62 (19.50)	27 (24.32)	35 (16.91)	0.137
Cardiovascular disease	92 (28.93)	25 (22.52)	67 (32.37)	0.07
Peptic ulcer	54 (16.98)	13 (11.71)	41 (19.81)	0.084
Chronic kidney disease	12 (3.77)	6 (5.41)	6 (2.90)	0.355
Hyperlipidemia	27 (8.49)	12 (10.81)	15 (7.25)	0.296
Autoimmune diseases	29 (9.12)	13 (11.71)	16 (7.73)	0.307
Psychiatric diseases	23 (7.23)	10 (9.01)	13 (6.28)	0.373
Alcohol drinking	20 (6.29)	13 (11.71)	7 (3.38)	0.006**
Betel nut chewing	70 (22.01)	43 (38.74)	27 (13.04)	<0.001**
Cigarette smoking	80 (25.16)	48 (43.24)	32 (15.46)	<0.001**
Periodontitis	111 (34.91)	37 (33.33)	47 (22.71)	0.458
TMJ disorder	15 (4.72)	5 (4.50)	10 (4.83)	>0.999
Candidiasis	40 (12.58)	22 (19.82)	18 (8.70)	0.007**
Skin lichen planus	13 (4.09)	1 (0.90)	12 (5.80)	0.039**
Cancer (other than oral cancer)	28 (8.81)	7 (6.31)	21 (10.14)	0.303
Oral cancer	33 (10.38)	23 (20.72)	10 (4.83)	<0.001*

Categorical variables are presented as numbers (%).

* *P* value was calculated using Fisher's exact test.

** *P* < 0.05, statistically significant; *P* > 0.05, statistically insignificant.

### The clinicopathological factors attributed to the development of oral cancer in OLP patients

The factors that influence the development of oral cancer in OLP patients are demonstrated in Table 2. After adjusting for confounders, patients with OPLs had a 2.98-fold (multivariable HR = 2.98; *P* = 0.035) higher possibility of developing oral cancer than those without OPLs. However, patients with periodontitis are not prone to develop oral cancer (multivariable HR = 0.68; *P* = 0.509). Patients above 50 years old (multivariable HR = 4.36; *P* = 0.005) showed a higher incidence of oral cancer than those below 50 years old. As far as gender is concerned, male OLP patients had a 4.37-fold higher occurrence of oral cancer than female OLP patients (crude HR = 4.37; *P* = 0.006), but the statistical data showed no significance after adjusting for all variables (multivariable HR = 2.36; *P* = 0.204). Besides, patients with underlying CKD (multivariable HR = 4.46; *P* = 0.017) and alcohol drinking habits (multivariable HR = 4.23; *P* = 0.011) also had higher risks of developing oral cancer.

**Table 2:** The influencing factors of oral cancer obtained after oral lichen planus in the current study.

Factors	Crude HR (95% CI)	*P* value	Multivariable HR (95% CI)^a^	*P* value
Periodontitis	1.11 (0.53, 2.34)	0.784	0.68 (0.22, 2.12)	0.509
Oral precancerous lesions	4.65 (2.21, 9.79)	<0.001*	2.98 (1.08, 8.21)	0.035*
Age				
<50	Ref.^b^		Ref.^b^	
≥50	3.15 (1.36, 7.29)	0.007*	4.36 (1.58, 12.08)	0.005*
Location				
Buccal	Ref.^b^		Ref.^b^	
Non-buccal	1.35 (0.63, 2.92)	0.442	1.48 (0.55, 4.00)	0.437
Male	4.37 (1.53, 12.43)	0.006*	2.36 (0.63, 8.86)	0.204
Liver diseases	0.66 (0.26, 1.72)	0.397	0.57 (0.18, 1.84)	0.349
Diabetes mellitus	1.56 (0.74, 3.28)	0.244	1.48 (0.55, 4.01)	0.437
Cardiovascular disease	1.11 (0.54, 2.25)	0.782	0.80 (0.29, 2.23)	0.664
Peptic ulcer	0.86 (0.36, 2.09)	0.742	1.48 (0.50, 4.42)	0.484
Chronic kidney disease	4.95 (1.90, 12.88)	0.001*	4.46 (1.30, 15.28)	0.017*
Hyperlipidemia	1.83 (0.75, 4.43)	0.182	1.42 (0.48, 4.21)	0.523
Autoimmune diseases	0.85 (0.26, 2.80)	0.793	0.59 (0.16, 2.95)	0.611
Psychiatric diseases	0.65 (0.16, 2.74)	0.561	0.35 (0.06, 2.10)	0.252
Alcohol drinking	3.71 (1.61, 8.55)	0.002*	4.23 (1.38, 12.93)	0.011*
Betel nut chewing	1.55 (0.76, 3.15)	0.230	2.02 (0.60, 6.82)	0.255
Cigarette smoking	1.59 (0.79, 3.20)	0.197	0.61 (0.18, 2.01)	0.414
TMJ disorder	1.61 (0.49, 5.28)	0.435	3.45 (0.78, 15.18)	0.102
Candidiasis	1.47 (0.60, 3.57)	0.400	0.77 (0.19, 3.17)	0.721
Skin lichen planus	2.01 (0.48, 8.42)	0.342	4.63 (0.49, 43.90)	0.182
Cancer (other than oral cancer)	1.68 (0.65, 4.37)	0.285	2.53 (0.81, 7.90)	0.110

HR, hazard ratio; CI, confidence interval; Ref, reference.

* *P* < 0.05, statistically significant; *P* > 0.05, statistically insignificant.

^a^ All variables were adjusted.

^b^ Reference as a control group within the group.

### The cumulative incidence rates of oral cancer in OLP patients

**Figure 1:**
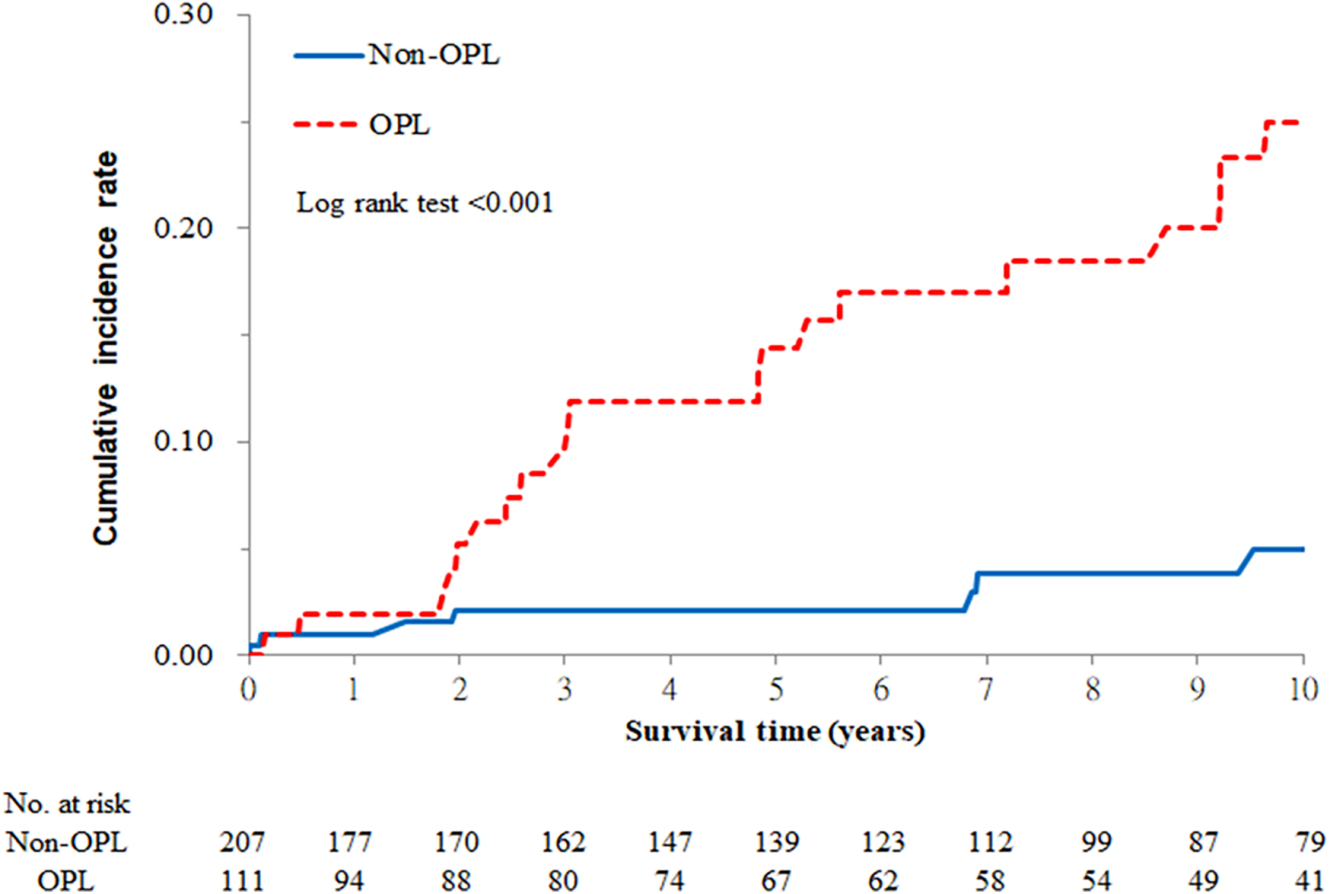
Ten-year cumulative incidence rate of oral cancer by oral precancerous lesions (OPLs).

**Figure 2:**
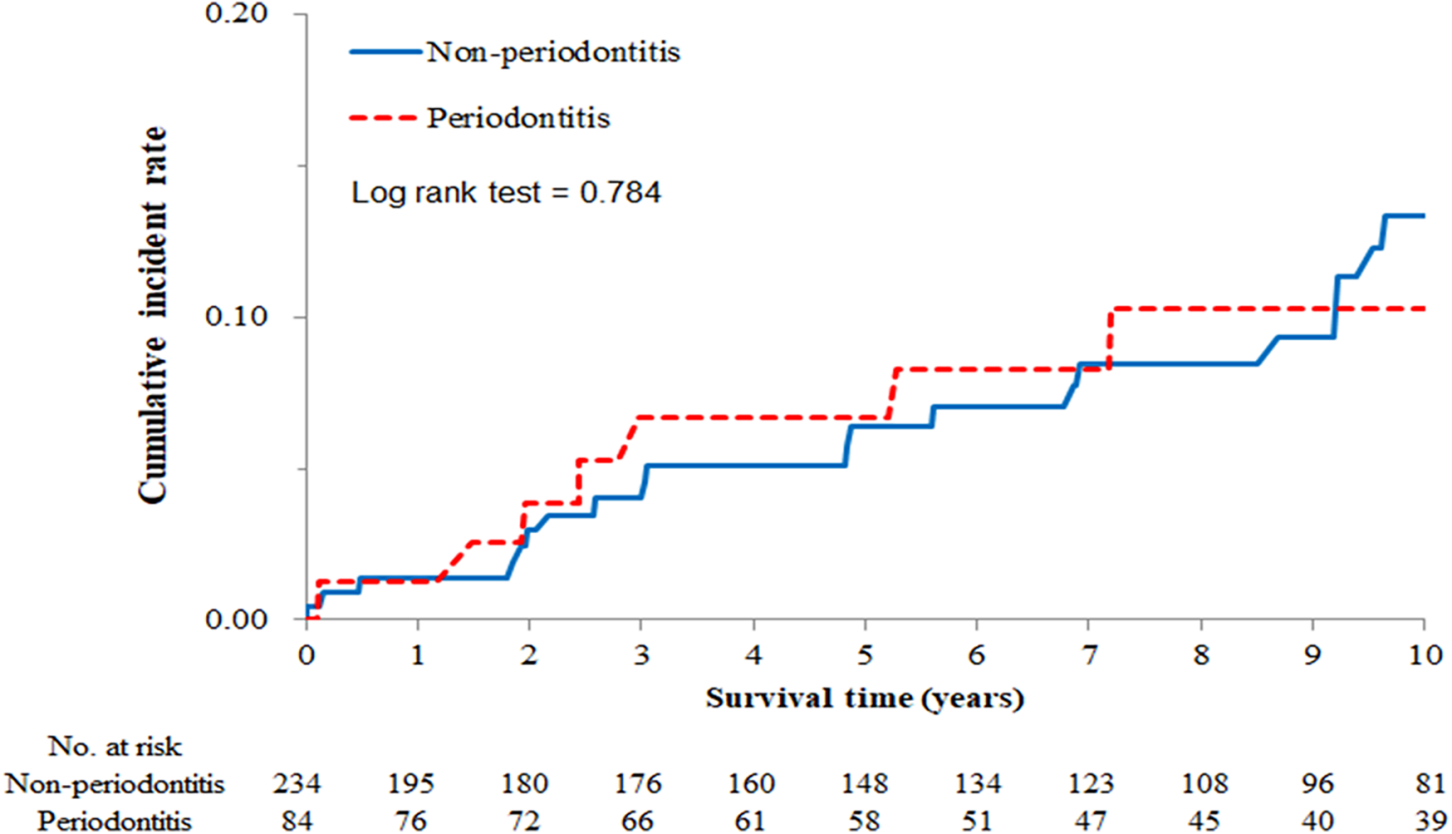
Ten-year cumulative incidence rate of oral cancer by periodontitis.

Since our study is a long-term longitudinal cohort, we can calculate the 10-year cumulative incidence rate of oral cancer in OPL patients and divide them according to different characteristics. After analyses, we found the 10-year cumulative incidence rate of oral cancer was higher in OLP patients with OPLs compared to those without (Log-rank test, *P* < 0.001) (Figure 1). However, this rate has no significant difference between OLP patients with and without periodontitis (Log-rank test, *P* = 0.784) (Figure 2).

## Discussion

Nowadays, OLP has been regarded as an oral potentially malignant disorder that can develop into oral cancer. The malignant transformation rate of OLP ranges from 0.44% to 2.28% (8). However, there has been no study focused on potential contributing factors, including age, sex, underlying diseases, and living habits simultaneously in the past 5 years.

In our previous study, risk factors for OLP patients who had higher risks of developing OPLs included periodontal disease, male, betel nut chewing, cigarette smoking, and candidiasis infection (5). After analyzing each patient's medical history and living habits, OLP patients with OPLs further showed higher rates of alcohol drinking habits and oral cancer occurrence. On the contrary, skin lichen planus is less common in OLP patients with OPLs, which is first indicated in this study (Table 1). Traditionally, lichen planus can be divided into mucosal subtypes (mainly inside the mouth, esophagus, and genitalia) and non-mucosal subtypes (manifest on the skin, scalp, and nails) (22). About 15% of OLP patients develop cutaneous lesions (23). However, there is currently no literature pointing out the correlation between skin lichen planus and OPLs. Notably, a systematic review and meta-analysis confirmed a strong association between lichen planus and signs of anxiety, whether in oral or cutaneous lichen planus (24).

In Table 2, OLP patients who are aged over 50, with OPLs, and have alcohol drinking habits were associated with an elevated risk of oral cancer. In addition, OLP patients with CKD demonstrated increased incidence rates of oral cancer. A large prospective cross-sectional study of 1,021 OLP patients in China showed that CKD stage and urinary albumin to creatinine ratio were positively correlated with OLP severity, which means CKD is a comorbidity in patients with OLP (25). And our cohort is the first to point out that CKD can be a risk factor for developing oral cancer in OPL patients. However, future multicenter studies and experiments to explore the underlying mechanism are essential.

As for OLP patients who had OPLs, the incidence of oral cancer remarkably increased after 2 years of the OPL diagnosis (Figure 1). In a retrospective cohort study based on the Taiwan National Health Insurance Database, the first quartile progression time to oral cancer diagnosis of oral leukoplakia (OLE), oral submucous fibrosis (OSF), and OLE + OSF were 29, 28.5, and 22 months, respectively (26). The time of developing oral cancer is similar to our OLP patients with OPLs.

Regarding periodontal disease, a Hungarian case-control study revealed a higher incidence of oral squamous cell carcinoma (OSCC) in patients with periodontitis (57.1%) compared to those without (28.6%) (27). Furthermore, a disproportionate number of oral cancer cases (72.1%) exhibited stage 4 periodontitis, while the control group predominantly (51.6%) presented with stage 2 periodontal disease, which figured out periodontitis to be an individual risk factor for oral cancer development (27). A review identified periodontitis as an inflammatory disorder caused by oral flora imbalance, and persistent mucosal inflammation and immune responses may contribute to malignant transformation (28). Recent evidence suggests that the subgingival microbiome and its metabolic byproducts can induce enduring changes in epithelial cells, potentially leading to carcinogenesis. For example, Porphyromonas gingivalis, a primary etiological agent in periodontitis, secretes proteases that act as signaling molecules, activating proteinase-activated receptors (PARs) to induce cell proliferation, apoptosis, cytokine production, autoimmunity, and inflammation. These proteases also degrade the extracellular matrix, damage host epithelium, and compromise the immune system, ultimately contributing to tumorigenesis. In addition to Porphyromonas gingivalis, Pseudomonas aeruginosa has also been implicated in OSCC carcinogenesis. This bacterium induces cellular alterations by synthesizing nitrous oxide, a process linked to elevated salivary nitric oxide levels observed in pre-cancerous conditions (28). However, in contrast to our previous findings linking OLP patients to a higher prevalence of chronic periodontitis, the present study did not reveal an increased incidence of oral cancer among OLP patients with periodontal disease (Figure 2).

Our study is mainly based on the oral cancer prevalence of OLP patients and its potential risk factors. Still, the molecular mechanisms underlying the transformation of OLP into oral cancer remain to need further elucidation. In a review focused on the hallmarks of cancer expression in OLP patients, one of the key molecular mechanisms driving epithelial cell proliferation is initiated by the binding of epidermal growth factor (EGF) to its receptor, epidermal growth factor receptor (EGFR), triggering a cascade of downstream hyperproliferative events (15). These events involve activating the MAPK and PI3K/Akt pathways, ultimately leading to upregulating genes that promote cell division, with CCND1/Cyclin D1 being particularly prominent. Moreover, the activation of these pathways can also occur through the stimulation of Ras protein and its downstream targets. Although *Ras* oncogenes are among the most frequently altered genes in OSCC, no primary studies are currently linking *Ras* to OLP. In addition, the PI3K/Akt pathway involves *PTEN* (its primary inhibitor, considered a tumor suppressor gene) and mTOR (which, upon activation, induces the expression of pro-proliferative oncogenes such as *MYC* and *CCND1*). However, only a few primary studies have explored the relationship between OLP and PTEN or mTOR (15). Overall, since the molecular mechanisms underlying the progression of OLP to malignancy have been investigated in a limited number of primary studies, further research is required to elucidate these mechanisms fully.

A recent meta-analysis reported that 1.1% of OLP lesions progress into OSCC with a higher incidence in smokers, alcohol users, and those infected with hepatitis C virus (29). While liver disease was not a significant factor in this study, these harmful living habits were strongly associated with the significant factors identified. In this study, 33 out of 318 OLP patients (10.38%) ultimately developed oral cancer, which is higher than the prevalence reported in current literature. This may be related to the fact that 34.9% (111 out of 318) of the study samples presented with OPLs. Additionally, the male proportion in our cohort was higher than that of females, differing from the higher prevalence of OLP in women reported in the literature. This discrepancy could be due to the higher proportion of males in southern Taiwan with habits such as smoking, alcohol consumption, and betel nut chewing.

Even though this is a long-term retrospective cohort study, some study limitations cannot be ignored. First, this is a single-institute retrospective study in southern Taiwan, which may lead to selection bias for not representative of patients in Asia and worldwide. Second, patients were included between 1995 and 2018 in our study, but there was no immunotherapy for oral cancer in Taiwan at that time, which may have affected patients' survival time. Third, although the number of patients we collected was as high as 318, 33 got oral cancer in the end, which may have caused research deviations. Lastly, the study was primarily focused on statistical analysis rather than underlying molecular mechanisms, which need further investigation.

## Conclusion

To our knowledge, this study is the first to report a lower prevalence of skin lichen planus among OLP patients with OPLs than those without OPLs. It was also the first to identify an increased risk of oral cancer among OLP patients with CKD. In conclusion, our findings reveal a significantly elevated risk of oral cancer among OLP patients with OPLs. Additionally, age of 50 years or older, CKD, and alcohol consumption emerged as independent risk factors for oral cancer in this patient population. However, periodontitis was not associated with an increased risk of oral cancer in OLP patients. Hence, for patients with OLP, healthcare providers should pay particular attention to the oral cavity when managing CKD, especially in elderly patients with a history of alcohol consumption and OPLs, as they are at increased risk for developing oral cancer.

## Data Availability

The original contributions presented in the study are included in the article/Supplementary Material, further inquiries can be directed to the corresponding authors.
